# A novel third mesh-like myometrial layer connects the longitudinal and circular muscle fibers -A potential stratum to coordinate uterine contractions-

**DOI:** 10.1038/s41598-020-65299-0

**Published:** 2020-05-19

**Authors:** Kyosuke Kagami, Masanori Ono, Takashi Iizuka, Takeo Matsumoto, Takashi Hosono, Naomi Sekizuka-Kagami, Yohei Shinmyo, Hiroshi Kawasaki, Hiroshi Fujiwara

**Affiliations:** 1 Department of Obstetrics and Gynecology, Graduate School of Medical Sciences, Kanazawa University, Kanazawa, Ishikawa Japan; 2 Department of Medical Neuroscience, Graduate School of Medical Sciences, Kanazawa University, Kanazawa, Ishikawa Japan; 3 Department of Health Sciences, Graduate School of Medical Science, Kanazawa University, Kanazawa, Ishikawa Japan

**Keywords:** Endocrine reproductive disorders, Reproductive biology

## Abstract

Periodic myometrial contraction is one of the important uterine functions to achieve embryo implantation and parturition. Although it is well-known that the mammalian myometrium is composed of longitudinal (outer) and circular (inner) layers, the precise mechanisms that coordinate both muscular contractions to produce peristaltic movements remain unclear. Recently, by treatment with our modified Clear Unobstructed Brain Imaging Cocktails and Computational analysis (CUBIC) tissue-clearing method, we obtained well-contrasted three-dimensional images of the transparent murine ovary using enhanced green fluorescent protein (EGFP) transgenic mice and light-sheet microscopy. Consequently, to investigate accurate anatomical connections between outer and inner myometrial fibers, we observed whole structures of the myometrium using a transparent murine uterus. By this method, we identified a novel muscle layer, a middle layer of the myometrium, which anatomically connects the conventional outer longitudinal and inner circular muscles. This new layer was visualized as a mesh-like structure and this structure was observed throughout the whole uterus from proximal to distal sites. In this area, CD31-positive vessels were abundantly localized around the mesh-like muscle fibers. In addition, CD34-positive uterine telocytes and tubulin β-3-positive nerve fibers were closely located in this middle layer. These findings indicate the presence of a novel mesh-like stratum that connects longitudinal and circular muscle layers, and suggest its coordinating role in myometrial contractions.

## Introduction

The uterus is a crucial reproductive organ for pregnancy and has several characteristics^1^. First, it houses the developing fetus, a semi-allograft of the mother, protecting the fetus from maternal immune attack^2^. Second, it enlarges during pregnancy to allow intrauterine fetal growth^3^. Third, it undergoes peristaltic contraction to achieve fetal delivery^4^. In general, the mammalian myometrium is composed of longitudinal (outer) and circular (inner) muscle layers. To flexibly adapt to fetal growth and adequately coordinate labor contraction, anatomical and functional communications between both muscle layers are important. Currently, inadequate uterine adaptation to fetal growth is known to lead to premature labor^5^, while abnormal peristaltic myometrial contraction is considered to cause dysmenorrhea^6^, endometriosis^7,8^, and infertility^9^. However, the precise mechanisms coordinating the functions of both muscle layers remain unknown.

To analyze the stereoscopic anatomy of reproductive organs, classical preparation of tissue sections and histological staining techniques have been performed. Although partial reconstruction of three-dimensional (3D) images based on these techniques is possible^10^, it is difficult to obtain whole 3D images of the uterus by a classical technique using sequential tissue sections alone. Recently, several groups developed excellent tissue-clearing methods such as Sca*l*eA2, See Deep Brain (SeeDB), CLARITY, 3D Imaging of Solvent-Cleared Organs (3DISCO), and CUBIC, and succeeded in producing various transparent tissues^11–15^. Combined with light-sheet laser scanning microscopy, these methods can provide clear 3D images of whole organs without preparing tissue sections^16^. Using a modified CUBIC tissue-clearing method, we also succeeded in making the pregnant murine uterus transparent and analyzing the specific distribution of the embryo-derived trophoblast that had invaded toward maternal uterine muscle layer^17^. Furthermore, we observed that EGFP transgenic mice had various ranges of cell-lineage-specific fluorescent activities, which enables us to create well-contrasted images^18^. Accordingly, we could obtain well-contrasted and 3D images of the whole ovary under light-sheet microscopy using a tissue-clearing technique and EGFP transgenic mice^18^.

Based on this advantage, we applied our modified CUBIC tissue-clearing method to the murine uterus to analyze the 3D structure of myometrium and elucidate the physiological mechanism of uterine contraction. Consequently, we identified a novel mesh-like muscle structure, a middle layer of myometrium, which anatomically connects the conventional outer longitudinal and inner circular muscle layers. Since this structure is one of the candidates to explain the mechanisms coordinating uterine peristaltic contractions, we further analyzed the mesh-like muscle region by immunohistochemical study together with 3D imaging under light-sheet microscopy.

## Results

### Tissue clearing of the non-pregnant uterus using the CUBIC method

**Figure 1:**
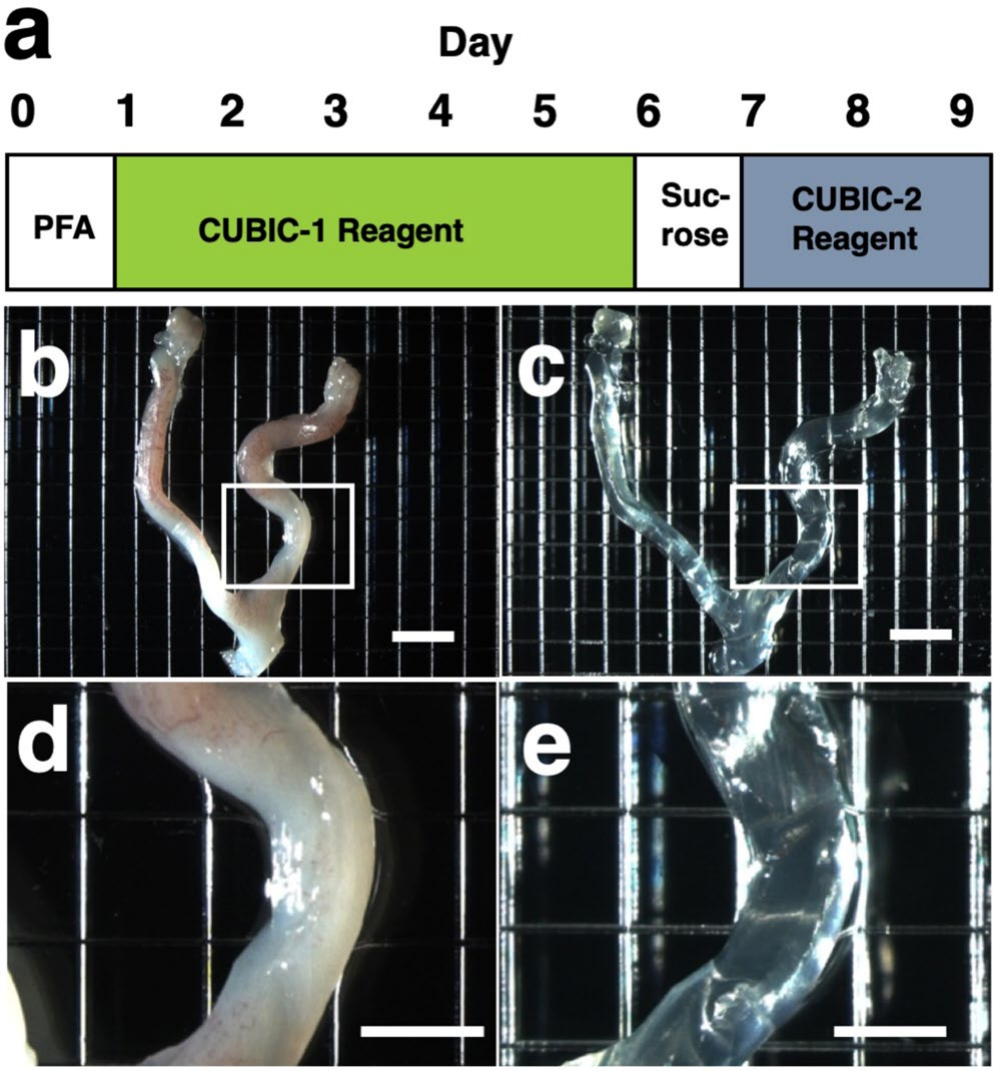
Tissue clearing of the uterus using CUBIC. (**a**) CUBIC protocol for the non-pregnant uterus. (**b**,**c**) Bright-field images of the uterus of wild-type adult mice before (**b**) and after (**c**) tissue clearing. (**d**) A highly magnified image within the white square of **(b**). (**e**) A highly magnified image within the white square of (**c**). Scale bars, 4 mm (**b,c**) and 2 mm (**d,e**).

Among several protocols for tissue clearing, we chose CUBIC, which has a marked advantage of efficient decolorization of endogenous chromophores within the tissues^15^. We previously reported that the pregnant uterus was effectively cleared by our modified CUBIC method. Non-pregnant female mice were fixed with a transcardial perfusion of 4% paraformaldehyde (PFA), and the uterus was isolated. In accordance with the CUBIC protocol, the isolated uterus was first immersed in CUBIC-1 reagent for 5 days, in 20% sucrose for 1 day, and subsequently in CUBIC-2 reagent for 2 days (Fig. 1a). Then, bright-field images were taken using a stereomicroscope.

As we previously observed in pregnant uterus, we found that the non-pregnant uterus became transparent using CUBIC (compare Fig. 1b,c). Importantly, the size of the uterus was not affected by CUBIC (compare Fig. 1d,e), although it was often reported that the size of organs became larger after tissue clearing^12^. These results suggest that CUBIC is an appropriate method for making the non-pregnant uterus transparent even though it contains thick myometrium.

### Detection of a novel mesh-like muscle layer in the myometrium using the modified CUBIC method and EGFP transgenic mice

To visualize fine structures in the uterus, we firstly combined CUBIC with propidium iodide (PI) nuclear staining (modified CUBIC method) and observed PI images by light-sheet microscopy. We successfully obtained not only sequential X-Y plane images but also angle-free cross-sectional images of the uterus with single-cell resolution without making tissue sections and detected fluorescence PI signals deep in the uterus (Supplementary Figure S1 and Supplementary Video S1).

**Figure 2:**
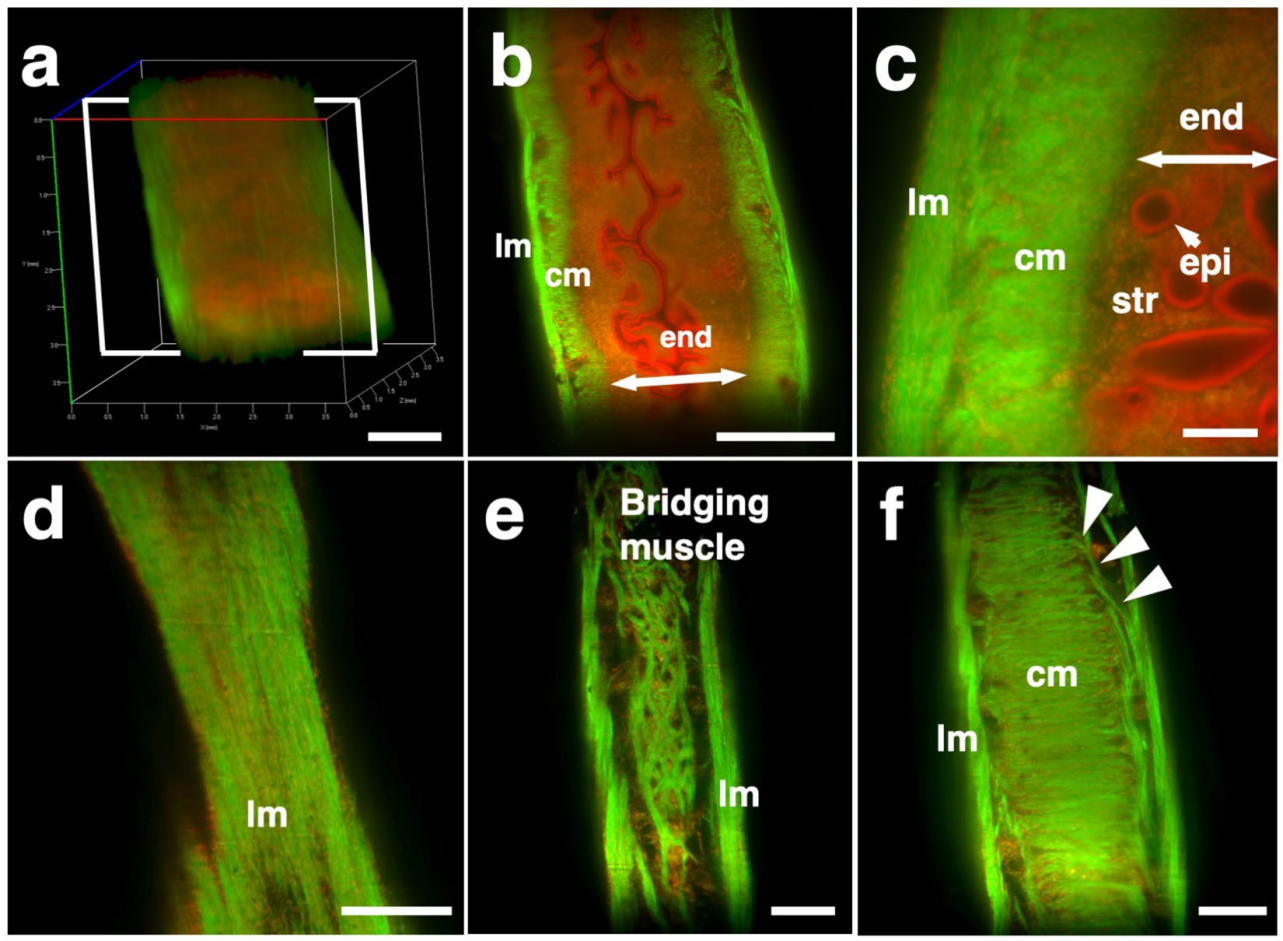
Three-dimensional and cross-sectional images of the EGFP-positive mouse uterus. Female CAG-EGFP transgenic mice were transcardially perfused with 4% PFA containing PI. After the isolated uterus was subjected to CUBIC, 3D and cross-sectional images were taken by light-sheet microscopy. (**a**) A 3D image of EGFP and PI signals of the uterus. (**b**) A sectional image within the white square of (**a**). (**c**) A highly magnified image of (**b**). (**d**–**f**) Reconstructed sequential coronal image of the myometrium showed an outer layer (**d**), middle layer (**e**), and inner layer (**f**). Note that EGFP fluorescence of the muscle fibers was high in the myometrium, but very low in the endometrium. A mesh-like structure of muscle fibers that connected the longitudinal and circumferential myometrium was observed in the middle muscle layer (arrowhead). lm, longitudinal muscle layer; cm, circumferential muscle layer; end, endometrium; str, stromal cells; epi, epithelial cells. Scale bars, 1 mm (**a**, **b**), 200 µm (**c**), and 500 µm (**d–f**).

Although PI nuclear staining clearly showed cellular distribution patterns, PI staining alone was not sufficient to recognize the shape of the cell body and/or histological structures of the uterus containing many myocytes, which have elongated cell bodies. Accordingly, we used transgenic mice expressing EGFP under the control of the CAG promoter, which contains the chicken beta-actin promoter and cytomegalovirus enhancer. The uterus of CAG-EGFP transgenic mice with PI staining was subjected to CUBIC, and 3D images were reconstructed by light-sheet microscopy (Fig. 2a). We clearly observed strong EGFP fluorescence in the myometrial layer in 2D images of X-Y cross-sections (Fig. 2b,c). Interestingly, although EGFP protein was clearly detected in both endometrial and myometrial layers (Supplementary Figure S2b and c), EGFP fluorescent signals were relatively weak in the endometrial component cells (Fig. 2c and Supplementary Figure S2a). This difference in EGFP signals facilitated recognition of the borderline between the endometrium and myometrium (Fig. 2b,c). The reconstructed images demonstrated fine structures of the EGFP-positive myometrial layers (Supplementary Video S2).

**Figure 3:**
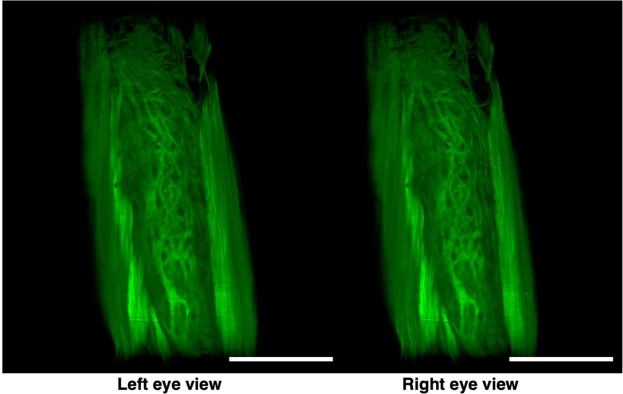
Stereoscopic image of the mesh-like muscle structure in the uterus of EGFP transgenic mice. Female CAG-EGFP transgenic mice were transcardially perfused with 4% PFA containing PI. After the isolated uterus was subjected to CUBIC, 3D images were taken by light-sheet microscopy, and then stereoscopic images were reconstructed. When producing a combined stereoscopic image from right-and-left 2D figures, we can clearly recognize an intricate whole picture of these mesh-like structures. Scale bars, 1 mm.

In rodents, it was reported that the myometrium consists of two muscle components, the outer longitudinal and inner circular muscle layers, and these two layers were separated by connective tissue and vasculature^19^. Immunohistochemical staining showed that EGFP fluorescence-positive cells co-expressed αSMA, showing that these EGFP-positive myometrial layers are composed of myocytes. Consequently, by our method, we clearly observed a novel bridging muscle layer, a middle layer of myometrium, which anatomically connected the longitudinal (Fig. 2d, lm) and circular muscle fibers (Fig. 2f, cm). We also found that these bridging muscles showed a mesh-like structure (Fig. 2e). The whole image of this novel muscle layer can be observed by 3D video, and this layer was demonstrated throughout the uterine tract (Supplementary Video S2). Furthermore, a stereoscopic image, which was produced from the datasets of 3D images, provided on an intricate picture of these mesh-like structures (Fig. 3).

### Immunohistochemical examination of a mesh-like muscle layer

**Figure 4:**
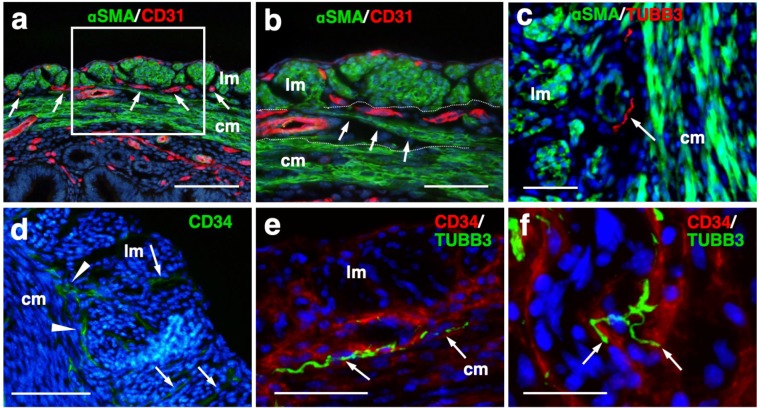
Immunohistochemical examination of the mesh-like muscle layer. Adult wild-type female mice were fixed with a transcardial perfusion of 4% PFA. Sections were stained with anti-CD31 (**a,b**), anti-TUBB3 (**c**,**e**,**f**), and anti-CD34 (**d–f**) antibody to investigate the cell populations in the mesh-like middle muscle layer. (**a**) CD31-positive endothelial cells (arrows) are abundant in the middle muscle layer. (**b**) A highly magnified image of the white square in (**a**). Muscle fibers (arrows) run across the vessel-rich region in the mesh-like layer (within the dotted lines). (**c**) Tubulin β-3 (TUBB3)-positive nerve axons were dominantly distributed within the mesh-like muscle layer. (**d**) CD34-positive myometrial telocytes that possess elongated thin telopods were predominant within the outer longitudinal layer (radially distributed, arrows) and the middle mesh-like layer (circularly distributed, arrowheads). (**e**) TUBB3-positive axonal fibers (arrows) were observed in parallel along the CD34-positive telocytes in the mesh-like muscle area. (**f**) These TUBB3-positive axonal terminals (arrow) were attached to CD34-positive telocytes. lm, longitudinal muscle layer; cm, circumferential muscle layer; αSMA, alpha smooth muscle actin; TUBB3, tubulin β-3. Scale bars, 100 µm (**a**), 50 µm (**b**–**e**), and 25 µm (**f**).

Although the 3D reconstructed uterine image showed the whole structures of bridging muscles, it could not provide us a detailed view of small vascular structures. Consequently, we additionally performed immunohistochemical analysis. CD31-positive blood vessels are abundantly observed in this area (Fig. 4a). Muscular fibers run across the vessel-rich region in the mesh-like layer (Fig. 4b).

In addition, the distribution of tubulin β-3 (TUBB3)-positive nerve axons was dominantly observed within the mesh-like muscle layer (Fig. 4c). Importantly, CD34-positive myometrial telocytes that possess elongated and thin telopods were predominantly observed within the outer longitudinal layer (radially distributed, arrows in Fig. 4d) and the middle mesh-like layer (circularly distributed, arrowheads in Fig. 4d), while its distribution is low within the inner circumferential muscles (Fig. 4d). Myometrial telocytes were functionally described as pace-making cells, which create homo- and heterocellular junctions with blood capillaries, nerve bundles, and muscle fibers^20,21^. TUBB3-positive axonal fibers were observed to run along the CD34-positive telocytes in the mesh-like muscle area (Fig. 4e). It was also shown that these TUBB3-positive axonal terminals attached CD34-positive telocytes (Fig. 4f).

## Discussion

Our modified CUBIC method combined with light-sheet microscopy successfully provided clear 3D images of the murine uterus. Myoglobin is one of the endogenous chromophores that interfere with the transparency of organs, and the uterus contains large amounts of myoglobin in the myometrial layer. Since aminoalcohol, which is one of main components of CUBIC reagent 1, is effective for the elution of myoglobin, we previously used the CUBIC method and succeeded in obtaining transparent images of the pregnant murine uterus^17^. Accordingly, we applied the CUBIC method to the non-pregnant uterus in this study, and confirmed that this method is also useful to make the non-pregnant uterus become transparent.

Interestingly, although a previous study reported that EGFP is expressed in almost all kinds of cells in CAG-EGFP transgenic mice^22^, our results showed that GFP fluorescence in the endometrium was relatively weaker than that of the myometrium in CAG-EGFP transgenic mice. To investigate the reason for the reduction of EGFP fluorescence activity, we examined immunohistochemical expression of EGFP in the uterus of CAG-EGFP transgenic mice. Contrary to EGFP fluorescence, EGFP immunoreactivity was strongly observed in the endometrial stromal cells and weakly in the endometrial epithelial cells (Supplementary Figure S2), suggesting that there are some differences in the efficiency of GFP gene expression under the control of the CAG promoter in reproductive organs.

Although the murine myometrium has been considered a two-layer structure^19,23^, we here identified a novel third muscle layer using EGFP transgenic mice, which anatomically connected the outer longitudinal and the inner circular muscles. This technique has the additional advantage of providing whole images of long continuous structures. Accordingly, although the murine uterus is cylindrical, the combination of computer-based free-angle and video observations using 3D image datasets enabled us to analyze the inner anatomical structures throughout the whole uterus. Consequently, we could confirm that mesh-like structures were present in the whole uterus from proximal to distal sites. Since the middle muscle layer connected longitudinal and circumferential myometrial fibers, this layer may play an important role in coordinating uterine contractions.

In human, the presence of middle vascular-rich layer, where muscle fibers are relatively scarce, was described previously^24^. Although no speculation about its role was provided, this area was also reported to contain mesh-like structure of muscle fibers. Since single human uterine body is embryologically developed by fusion of bilateral müllerian ducts, it is reasonable that the communication of muscle fibers between right and left müllerian ducts becomes manifested as mesh-like structures. However, considering that the rat/mouse müllerian ducts are not fused and remain as bilateral uterine bodies, human middle layer can correspond to the fusion area of the third muscle layer in the müllerian ducts, which was realized by this study. Consequently, the further investigation of the murine third muscle layer may contribute to clarifying the physiological roles of this middle layer in human uterus.

In this area, CD31-positive vessels were localized around the mesh-like muscle fibers. Recently, using a multi-electrode array recording system, Lutton *et al*. demonstrated that electrical potentials in the pregnant rat uterus are initiated in distinct myometrial bundles that bridge the longitudinal and circular muscle layers, which are located in the placental bed of individual implantation sites. They also reported that these muscle bundles had not been previously identified and they bridged across blood vessels located between longitudinal and circumferential muscle layers^25^. In this study, we observed muscular fibers running across a vessel-rich region in the mesh-like layer. The common anatomical characteristics suggest that the muscle fibers in the mesh-like layer of the murine uterus correspond to the electrical potential-initiating muscle bundles of the pregnant rat uterus. Notably, co-localization of CD34-positive uterine telocytes, which were proposed as pace-making cells^20^, and TUBB3-positive nerve fibers was dominantly identified in this middle layer, especially near the boundary of the outer layer. Furthermore, double-staining immunohistochemistry confirmed the close contact between telocytes and nerve fibers, suggesting that these areas are potentially regulatory centers that initially receive signals from the automatic nervous system and send them to myometrial cells via telocytes.

Importantly, since telocytes express estrogen and progesterone receptors and can respond to steroid hormone stimulation^26^, they were proposed to act as sensors of sex hormone levels^27^. Based on these findings, we propose that the novel mesh-like third muscle layer is involved in the control of autonomic uterine contraction in the presence of sex hormones. Since telocytes were also reported to contact capillaries^27,28^, this region may regulate blood flow within the myometrium. To confirm this, further analyses involving an electro-physiological study and calcium imaging should be performed.

## Methods

### Preparation of reagents

CUBIC reagents were prepared as described^15^. CUBIC-1 reagent was prepared as a mixture of 25% weight/weight (w/w) urea (Nacalai Tesque, 35904-45, Japan), 25% weight/volume (w/v) N, N, N′, N′-tetrakis (2-hydroxypropyl) ethylenediamine (Tokyo Chemical Industry, T0781, Japan), and 15% (w/v) polyethylene glycol mono-pisooctylphenyl ether (Triton X-100) (Nacalai Tesque, 25987-85, Japan). CUBIC-2 reagent was prepared as a mixture of 50% (w/v) sucrose (Nacalai Tesque, 30403-55, Japan), 25% (w/v) urea, 10% (w/v) 2, 20, 20′-nitrilotriethanol (Wako, 145-05605, Japan), and 0.1% volume/volume (v/v) Triton X-100. Both reagents were prepared just prior to use. Before adding Triton X-100, all other chemicals were dissolved with a hot stirrer at 60 °C. Distilled water was added during a mixing step in order to compensate for water evaporation. After all chemicals except Triton X-100 were dissolved, the solution was cooled to room temperature, and finally Triton X-100 was added.

### Animals

We used nine transgenic female mice expressing EGFP under the control of the CAG promoter (C57BL/6-Tg)^22^ and ten wild-type female mice (CD-1/ICR). CAG-EGFP mice were sacrificed at the age of 6–12 months and wild-type mice were the age of 3–4 months. Wild-type mice were purchased from SLC (Hamamatsu, Japan), and all mice were reared under a normal 12-hour light/dark schedule. All experimental procedures and housing conditions were approved by the Animal Care and Use Committee of the Kanazawa University Animal Experiment Committee, and all of the animals were cared for and treated humanely in accordance with the Institutional Guidelines for Experiments Using Animals.

### The CUBIC protocol for the uterus

CUBIC was performed as previously described^15^ with modifications that we reported^17,18^ (Fig. 1a). After deep anesthesia with pentobarbital, pregnant mice were fixed by transcardial perfusion using 4% PFA/PBS and PI (Life Technologies, 10 mg/mL solution), and then the uteri were isolated. The isolated uteri were further immersed in 4% PFA at 4 °C overnight. Then, the fixed organs were immersed in CUBIC-1 reagent at 37 °C for 3 days with gentle shaking. After CUBIC-1 reagent was changed, the organ was immersed for 2 additional days. The organ was washed with PBS 3 times at room temperature with gentle shaking, immersed in 20% sucrose in PBS for one day, and immersed in CUBIC-2 reagent for 2 days. For immersion staining with PI, 10 mg/mL PI was added to CUBIC-1 reagent.

### Immunostaining

Adult female mice were deeply anesthetized and transcardially perfused with 4% PFA in PBS as previously described^29^. To make sections, the uterus was partially dissected, post-fixed by overnight immersion in the same fixative, cryoprotected by overnight immersion in sucrose-containing PBS, and embedded in Optimal Cutting Temperature (OCT) compound (Sakura Finetek, Japan)^17^. Sections of 14-μm thickness were made using a cryostat, permeabilized with 0.5% Triton X-100 in PBS, and incubated at 4 °C overnight with first antibody^17,18^. After being incubated at 37 °C for 2 hours with Alexa 488- or Cy3-conjugated secondary antibody and 1 μg/mL Hoechst 33342, the sections were washed and mounted with Mowiol (Sigma-Aldrich)^17,18^. Antibodies used for immunostaining were as follows: rabbit anti-green fluorescent protein (GFP) antibody (Molecular Probe A-11122, 1:500), rat anti-CD31 antibody (BD Pharmingen 550274, 1:500), rat anti-CD34 antibody (Abcam ab8158, 1:500), rabbit anti-alpha smooth muscle actin (αSMA) antibody (Abcam ab5694, 1:200), rabbit anti-tubulin β-3 (TUBB3) antibody (BioLegend PRB-435P, 1:500), and Alexa488 conjugated mouse anti-tubulin β-3 (TUBB3) antibody (BioLegend A488-435L, 1:500).

### Microscopy and image analysis

Image analysis was performed as we described previously^17,18^. Bright-field images of the uterus were taken using a stereomicroscope (MZ16F, Leica). Tissue sections were examined with an epifluorescence microscope (BZ-X710, Keyence). Three-dimensional images of transparent organs were acquired using a light-sheet microscope (Lightsheet Z.1, Carl Zeiss)^17,18^. Images of the uterus were obtained using a 5×/0.16 NA objective lens, and detailed single-cell resolution images were acquired using a 20×/1.0 NA objective lens for the clearing method. Three-dimensional images were analyzed using ZEN software (Carl Zeiss)^17^.

## Supplementary information

Supplementary Figure S1

Supplementary Figure S2

Supplementary Video S1

Supplementary Video S2
