# Supplementary figures and images for: A novel third mesh-like myometrial layer connects the longitudinal and circular muscle fibers -A potential stratum to coordinate uterine contractions-
Source: Sci Rep. 2020 May 19;10:8274. doi: 10.1038/s41598-020-65299-0 (PMC7237439; doi:10.1038/s41598-020-65299-0)

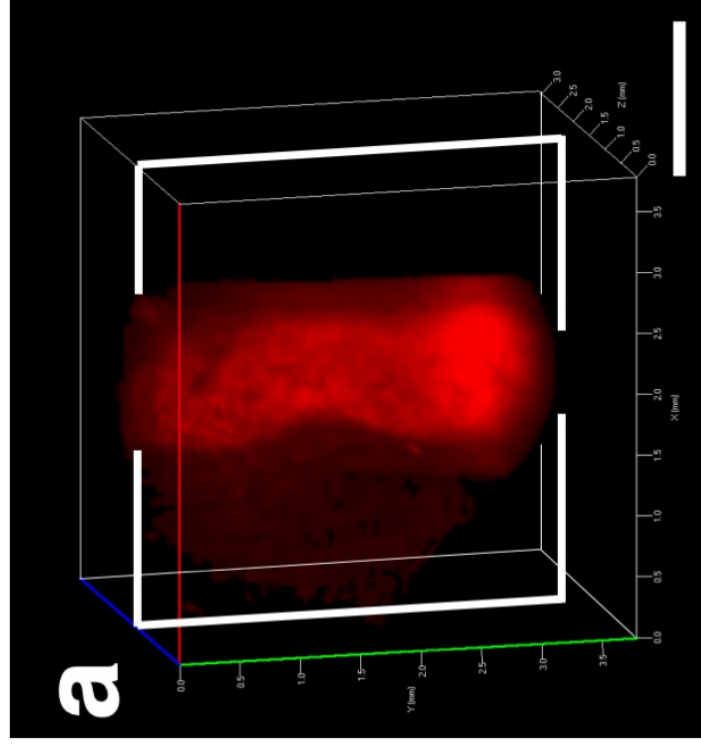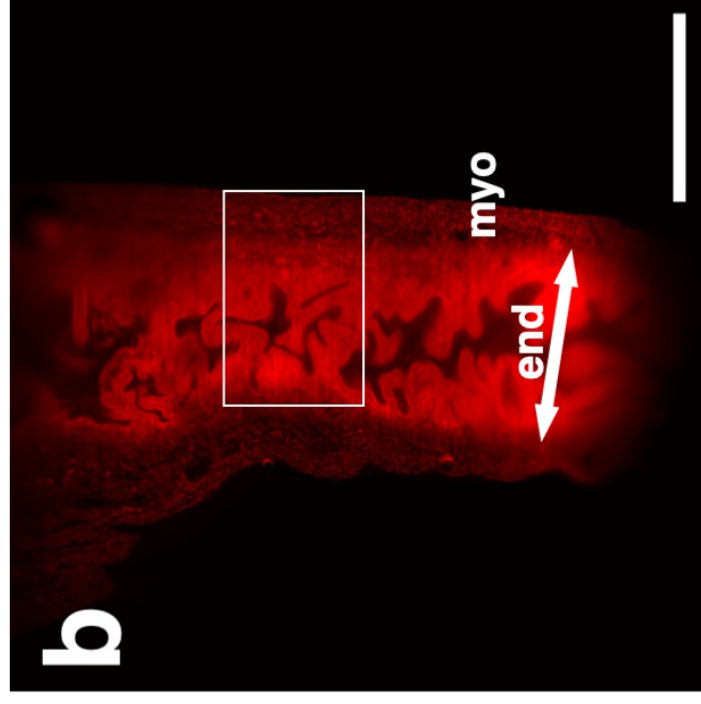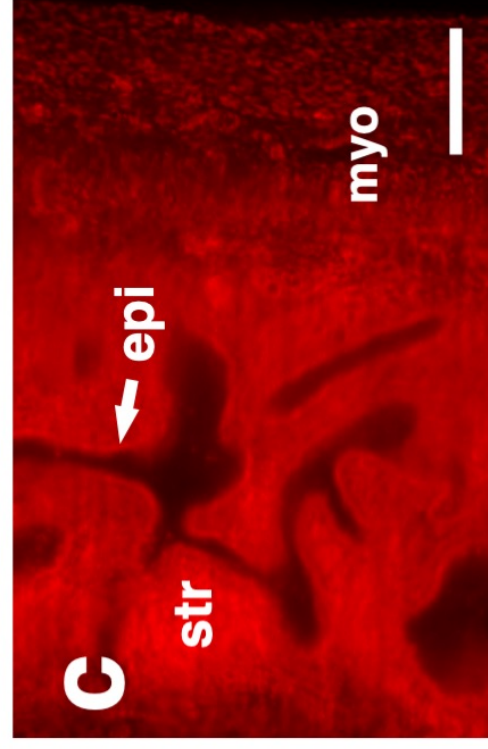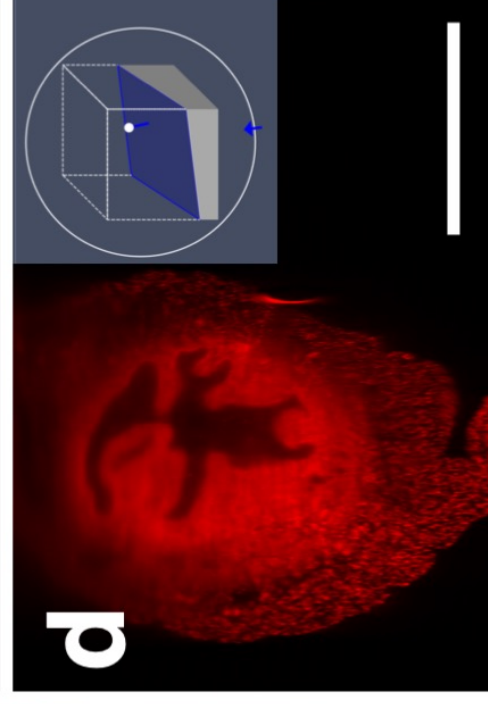

Supplementary Figure S1, Kagami et al.

Supplement: Supplementary file 1 — Supplementary Figure S1 [file 41598_2020_65299_MOESM1_ESM.pdf]

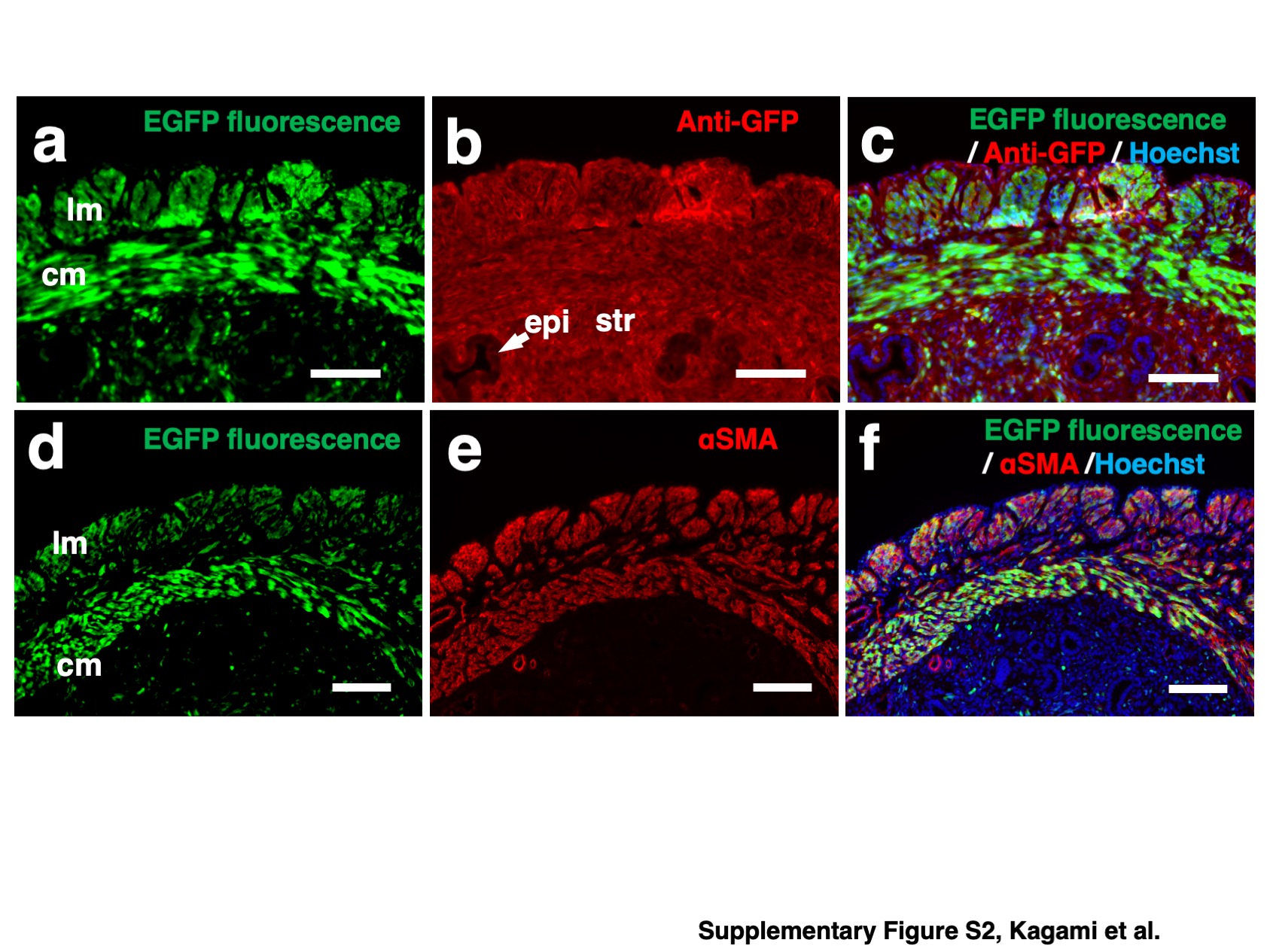

Supplement: Supplementary file 2 — Supplementary Figure S2 [file 41598_2020_65299_MOESM2_ESM.jpg]
